# Formation of Recalcitrant Compounds during Anaerobic Digestion of Thermally Pre-Treated Sludge: A Critical Macromolecular and Structural Study

**DOI:** 10.3390/ijerph20010558

**Published:** 2022-12-29

**Authors:** Eduardo Ortega-Martínez, Rolando Chamy, David Jeison

**Affiliations:** 1 Escuela de Ingeniería Bioquímica, Pontificia Universidad Católica de Valparaíso, Avenida Brasil 2085, Valparaíso 2362803, Chile; 2 Núcleo Biotecnología Curauma, Pontificia Universidad Católica de Valparaíso, Avenida Universidad 330, Valparaíso 2373223, Chile

**Keywords:** anaerobic digestion, thermal hydrolysis, pre-treatment, humic-like substances, FTIR

## Abstract

Thermal hydrolysis, when used as pre-treatment, enhances the anaerobic digestion of sewage sludge; moreover, due to the high temperature normally applied, undesirable recalcitrant compounds via Maillard reactions may also be formed. However, although the appearance of these recalcitrant compounds is widely reported, more information on the formation, structure, and fate of these compounds is still needed. This study was focused on understanding the amount and whereabouts of such compounds during the anaerobic digestion process with thermal pre-treatment in soluble and total phase and advance in its structural identification by analyzing their infrared (IR) spectra. It was found that, even with the improved methane production and COD degradation, at 165 °C for 30 min, humic-like compounds are formed which could not be degraded at the anaerobic digestion step. These compounds account for 25% of the original sludge. Infrared spectroscopy proved to be a powerful technique, permitting their differentiation from the natural humic-like compounds. This research provides new information about the structure of melanoidins at every stage of the thermal hydrolysis pre-treatment and how they contribute to the dissolved organic nitrogen.

## 1. Introduction

Waste-activated sludge (WAS) is traditionally treated by conventional anaerobic digestion (CAD) processes. The use of the CAD process is attractive not only due the amount of sludge reduction it can provide, but also because of biogas production. Due to the complex nature of WAS, hydrolysis is normally the limiting step during anaerobic conversion, determining the dynamics of the whole process [1,2]. To overcome this limitation, a series of pre-treatments have been proposed [3]. The addition of a thermal hydrolysis (TH) step before the anaerobic digestion of WAS is one of the most convenient alternatives. It has proven to be efficient, not only at lab scale, but also at full scale [4,5,6,7]. In a typical TH-AD process, the sludge is submitted to temperatures within the range of 140–180 °C for 20 to 40 min [3,7,8,9,10]. Under these conditions, high levels of hydrolysis and COD solubilization are achieved, resulting in well-documented advantages, such as (i) an increase in methane production and COD biodegradation, (ii) the enhancement of digested sludge dewaterability, and (iii) pathogen reduction. However, this process is not exempt of disadvantages, such as (i) the inhibition of the nitrogen removal processes (e.g., annamox), (ii) possible recalcitrant compounds’ formation, (iii) production of colored compounds affecting downstream process such as UV disinfection [6,9,11]. Many researchers agree that melanoidins formed by Maillard reactions are responsible for these drawbacks [6,9,11]. Melanoidins are brownish complex compounds of high molecular weight, formed at the final stage of Maillard reaction [3,9,12,13], and are difficult to degrade by aerobic and anaerobic means [3,9]. It has been reported that these compounds share similarities with humic acids, to the point of being called “artificial humic acids” or “humic-like compounds” [6,14]. However, their structure is still not fully known, making their identification and quantification a difficult task [14].

Several analytic techniques have been used to characterize and quantify these compounds, such as UV-Vis, fluorescence spectroscopy (emission/excitation matrix), infrared (IR) spectroscopy, and nuclear magnetic resonance (NMR) spectroscopy, among many others [15]. However, IR spectroscopy has gained increasing attention. In our previous research, we identified that the melanoidins formed during thermal treatment of a simple mixture of glucose and glycine presented a characteristic increase in the peaks associated with amide II bonds in the IR spectrum [16]. However, it is not clear if the same could be expected for sludge, considering the complex interactions that may occur. To the authors’ knowledge, there is no study characterizing each stage of the TH-AD of sludge using IR spectroscopy.

Some researchers have evidenced the formation of melanoidins during the TH-AD process. Penaud et al. (2000) [17] observed that the thermal treatment of industrially spent microbial biomass resulted in the formation of melanoidins, which, in turn, decreased its biodegradability. Bougrier et al. (2007) [18] attributed the coloration of the liquid fraction of the digested sludge at 190 °C to melanoidins formation. Dwyer et al. (2008) [19] found that a reduction in temperature from 165 °C to 145 °C could reduce the production of melanoidins, leaving the sludge digestibility unaffected. Moreover, Lu et al. (2018) [20] found that heterogeneous polymers of high molecular weight (melanoidins) were formed during the thermal pre-treatment of sludge at 172 °C and were only slightly degraded at the anaerobic digestion step. Although many authors have reported the appearance of recalcitrant compounds resulting from thermal pre-treatment [9], more information on the formation and fate of these compounds is still needed.

The aim of this study is to advance in the understanding of the production and whereabouts of recalcitrant compounds formed during the anaerobic digestion of WAS, when thermal pre-treatment is provided, by quantifying them in each step of the process and following changes in sludge structure via IR spectroscopy.

## 2. Materials and Methods

### 2.1. Substrates

Thermally pre-treated and thickened activated sludge was obtained from Mapocho/Trebal wastewater treatment plant (WWTP), located in Santiago, Chile. This is a full scale WWTP equipped with a CAMBI THP process, providing a thermal pre-treatment at 165 °C for 30 min. Non-pre-treated and thickened activated sludge was also obtained from the same WWTP. A summary of the characterization of each sludge is show in Table 1.

### 2.2. Operation of the TH-AD and CAD Digesters

**Figure 1:**
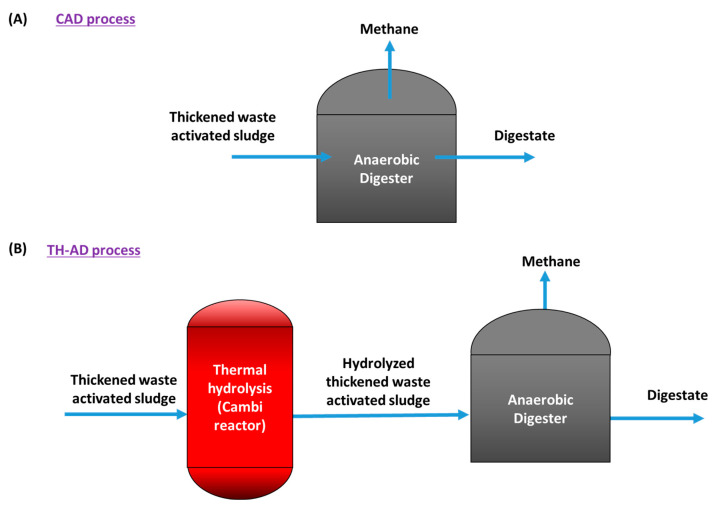
Schematic view of (**A**) CAD process and (**B**) TH-AD process.

Two anaerobic digesters of 1 L of working volume were operated. The first one, the TH-AD digester, was fed with thermally pre-treated and thickened activated sludge. The second one, the CAD digester, was fed with thickened activated sludge and was used as control. The conditions used were 25 days of hydraulic retention time (HRT) and an organic loading rate (OLR) of 3.4 g VS L^−1^ day^−1^ for the TH-AD digester and 33 days of HRT and an OLR of 3.0 g VS L^−1^ day^−1^ for the CAD digester. Both digesters were inoculated with anaerobic sludge obtained from the Mapocho/Trebal WWTP. The TH-AD digester was inoculated with a sludge fed with thermally pre-treated, waste-activated sludge, and the CAD digester was inoculated with a sludge fed with waste-activated sludge. The solids content of the inoculum were 37.3 g TS L^−1^ and 23.8 g VS L^−1^ for the TH-AD digester, and 16.1 g TS L^−1^ and 10.0 g VS L^−1^ in the case of CAD digester. A schematic view of the process is provided in Figure 1.

A summary of the operational conditions can be found in the supplementary materials (see Table S1). Both digesters were operated for 260 days and fed on a daily basis. CODt, CODs, and VS degradation during the operation of the digesters were calculated as described by Koch (2015) [21].

### 2.3. Anaerobic Biodegradability of Digestates

Biodegradability of the digestates was determined, as an estimate for the remaining biodegradable matter, after the anaerobic digestion step (with and without pre-treatment). Biodegradability was measured through BMP assays without the addition of any external substrate. Tests were performed using the methodology described elsewhere [22]. The biodegradability was calculated according to the following equation:

(1) $$\mathrm{Biodegradability}\left(\%\right)=100\times \frac{{\mathrm{Standarized}\;\mathrm{methane}\;\mathrm{production}\;\mathrm{in}\;\mathrm{ml}\;\mathrm{CH}}_{4}{\;g\;\mathrm{COD}}^{-1}\;}{350{\;\mathrm{mL}\;\mathrm{CH}}_{4}{\;g\;\mathrm{COD}}^{-1}\;}$$

where methane production is expressed under standard conditions. The value 350 mL g COD^−1^ represents the maximum theoretical methane production, as described by Angelidaki and Sanders (2004) [22].

### 2.4. Analytical Methods

Total and volatile solids (TS and VS, respectively) were measured by the methods described in APHA (2005) [23]. Alkalinity and VFA were measured by the method described by Ripley et al. (1986) [24]. Total ammoniacal nitrogen was measured using the salicylate method, using the Nitrogen Ammonia Reagent Set, High Range Test ‘N Tube™ AmVer™ kits from Hach (Loveland, CO, USA). Color was recorded in platinum–cobalt (Pt-Co) units, as described by Dwyer et al. (2008) [19]. Biogas production was determined using RITTER milligas counter apparatus. Methane content in the biogas was analyzed using a gas chromatograph (Clarus 500, Perkin Elmer, Waltham, MA, USA), equipped with a thermal conductivity detector (TCD) and a molecular sieve column (Molesieve 5A, mesh 80/100, 4 m × 1/8 in., Agilent, Santa Clara, CA, USA). The temperatures of the column and the TCD detector were 70 °C and 120 °C, respectively, and helium was used as the carrier gas at a flow rate of 15 mL min^−1^. Methane production is reported considering the standard conditions (0 °C and 1 atm). Total COD (CODt) was measured according to the method 5220 D described in APHA (2005) [23]. Carbohydrates (C) were measured using the phenol/sulphuric acid method, described by Le and Stuckey (2016) [25]. Hydrazine sulphate was added to the sulphuric acid to avoid interferences [26]. Proteins (P) and humic-like compounds (H-LC) were measured according to a modification of the Lowry method proposed by Frølund et al. (1995) [27]. Soluble proteins, humic-like compounds, carbohydrates, and COD were measured by applying the described methods to the soluble fraction of the samples, filtered using a 0.2 µm pore sized syringe filter.

Fourier-transform infrared (FTIR) analysis was used as a tool to identify functional groups that may belong to recalcitrant compounds. Samples of digestates coming from both CAD and TH-AD processes were analyzed. Samples were freeze dried, up to 4 ± 1% of humidity. Then, the spectra were acquired with a JASCO FT/IR-4600 spectrometer, using diamond single reflection attenuated total reflectance (FTIR-ATR). Spectra were obtained with 64 scans per spectrum, at a spectral resolution of 0.5 cm^−1^, in the wavenumber range from 4000 to 600 cm^−1^. Baseline correction and scaling normalization were made in the Wiley KnowItAll^TM^ Software 2021 (version 21.1.91). To promote a better understanding of the peaks position and intensity, spectral analysis was conducted. Deconvolution was made using Origin 2019 PRO software by modelling the spectra to a Lorentz profile.

Student’s *t*-tests were used to determine statistical differences between carbohydrates, humic-like compounds, and protein concentrations. The same test was also used to establish differences between COD, CODs, methane production, color, total ammoniacal nitrogen and others.

## 3. Results

### 3.1. Operation of TH-AD and CAD Digesters

Table 2 shows a summary of the several operation parameters, characterizing the operation of CAD and TH-AD digesters. CAD digester removed 47% of VS, 42% of CODt, 24% of CODs, and produced around 230 mL CH_4_ L digester^−1^ d^−1^. On the other hand, TH-AD digester removed close to 50% of the VS, 60% of CODt, 70% of CODs, and produced around 770 mL CH_4_ L digester^−1^ d^−1^. The VFA/Alkalinity ratio remained below 0.3 during the operation of both digesters. Color, on the other hand, was way higher in the TH-AD digester in comparison to CAD digester. The high concentration of total ammoniacal nitrogen (TAN) in the digestates was also observed in both digesters. However, the TH-AD process had a higher TAN yield than the CAD process.

The amount of anaerobically biodegradable organic matter remaining after each process was assessed by using BMP assays. Only 22 ± 1% and 25 ± 1% of the organic matter present in the digestates was anaerobically biodegradable for TH-AD and CAD, respectively. Similar kinetics of biodegradation were obtained for both digestates, with the TH-AD digestate showing only a slightly faster rate (supplementary materials Figure S1).

### 3.2. Analysis of Macromolecules in CAD and TH-AD Processes

**Figure 2:**
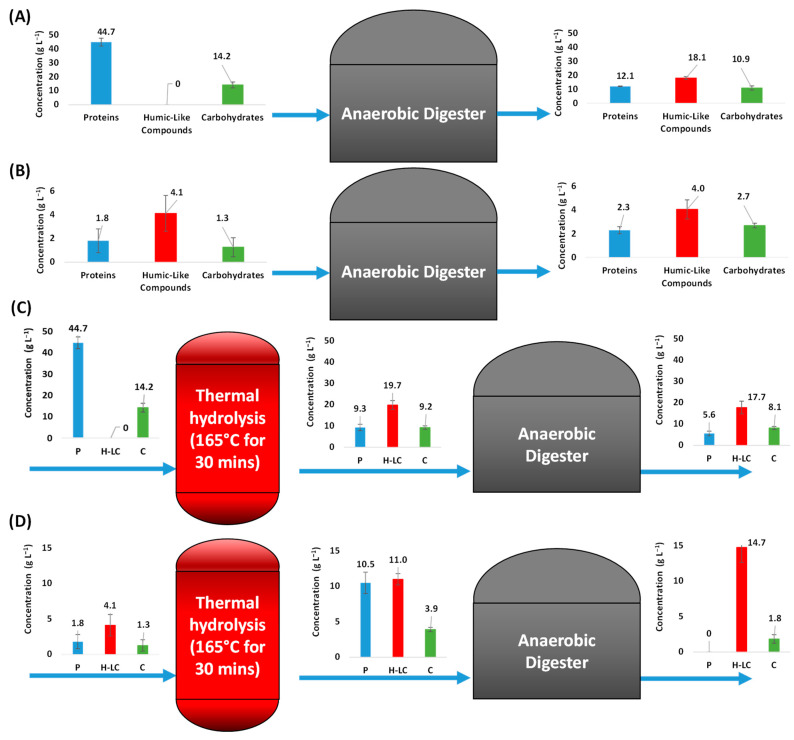
Concentration of (**A**) total and (**B**) Soluble proteins (P), carbohydrates (c), and humic-like compounds (H-LC) on inlet and outlet of anaerobic digester during CAD process and concentration of (**C**) total and (**D**) soluble P, C, and H-LC in each stage of the TH-AD process. Dilution effects due to steam addition were not considered. Error bars represent the standard deviation presenting samples taken at the steady state.

Figure 2A,B shows total and soluble concentrations of carbohydrates, proteins, and humic-like compounds at the inlet and outlet of the CAD process. Moreover, 73% of total proteins (from 44.7 g L^−1^ to 12 g L^−1^) and 23% of total carbohydrates (from 14.2 g L^−1^ to 10.9 g L^−1^) were degraded by the anaerobic digestion process. On the other hand, humic-like compounds increased from 0 to 18.1 g L^−1^. When observing soluble fraction compositions, little or no variations were observed.

Figure 2C,D shows total and soluble carbohydrates, proteins, and humic-like compounds at different stages of the TH-AD process. As a result of thermal pre-treatment, total proteins and carbohydrates decreased by 79% (from 44.7 g L^−1^ to 9.3 g L^−1^) and 35% (from 14.2 g L^−1^ to 9.2 g L^−1^), respectively. Total humic-like compounds increased from 0 to almost 20 g L^−1^. Soluble carbohydrates and humic-like compounds increased by around three times and soluble proteins increased by around six times.

TH-AD digester performance shows that 40% of the total proteins and 12% of the total carbohydrates were degraded. Data also shows a 100% and 53% reduction in soluble proteins and carbohydrates, respectively. However, only 10% of the total humic-like compounds that were formed at the pre-treatment step were degraded. Considering the dilution due to the steam addition (46% in our study), 0.24 kg of total humic-like compounds were produced per kg of volatile solids of the original thickened activated sludge entering the TH-AD process. Moreover, soluble humic-like compounds increased 34% (from 11.0 g L^−1^ to 14.7 g L^−1^).

### 3.3. Fourier-Transform Infrared Spectroscopy Analysis

**Figure 3:**
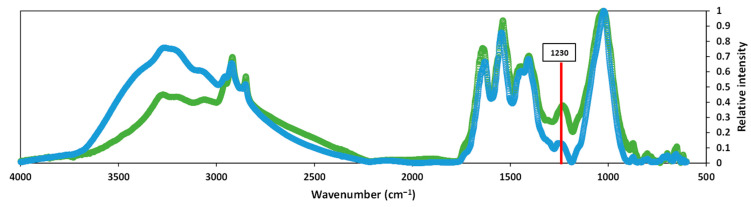
FTIR spectra of the feed (green lines) and effluent of CAD digester (blue lines).

FTIR spectra was determined for the different stages of the CAD and TH-AD processes. For peak assignments, the following references were used: [28,29,30,31,32,33,34,35,36,37,38]. Figure 3 shows the IR spectra of the thickened activated sludge before and after digestion in the CAD digester. Although many changes occurred, two major ones can be appreciated: a reduction in the peak at 1230 cm^−1^, which is attributed to the peptides and proteins degradation [29] and an increase in the intensity of the peaks between 3000 cm^−1^ and 3600 cm^−1^, the changes of which were attributed to N-H and/or O-H stretching [28].

**Figure 4:**
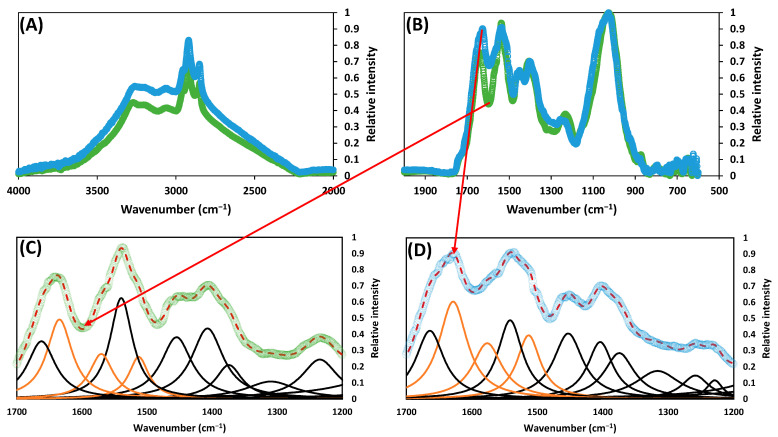
FTIR of the sludge before (green lines) and after (blue lines) thermal treatment, during TH-AD process, between (**A**) the range of 4000 cm^−1^ and 2000 cm^−1^ and (**B**) between the range of 2000 cm^−1^ and 600 cm^−1^. The deconvoluted spectrum with the fitted component band is also shown for (**C**) the untreated sludge and (**D**) the pre-treated sludge, between the range of 2000 cm^−1^ and 600 cm^−1^. Orange lines represent the fitted curves associated with recalcitrant compounds (peaks at 1630 cm^−1^, 1580 cm^−1^, and 1510 cm^−1^) and black lines represent the other fitted curves between the range of 2000 cm^−1^ and 600 cm^−1^.

On the other hand, when the thermal pre-treatment was applied, peaks increased in the range between 4000 cm^−1^ and 2000 cm^−1^ (see Figure 4A). Further analysis, by deconvoluting the curve, showed that these peaks were centered at 3390 cm^−1^ (N-H and/or O-H stretching) and 2890 cm^−1^ (C-H stretching) [28], which is attributed to the solubilization of proteins (see supplementary materials Figure S2). Additionally, between 2000 cm^−1^ and 600 cm^−1^, the increase in three peaks was detected (see Figure 4B–D). The peak centered at 1630 cm^−1^ was attributed to the ν(C=N) of the imine in the Schiff base [30,31]. The peaks centered at 1580 cm^−1^ and 1510 cm^−1^ were attributed to COO- bonds [36] and C-N stretching and/or the N-H bending of amide II bonds [30,32,33,34,35,37,38].

**Figure 5:**
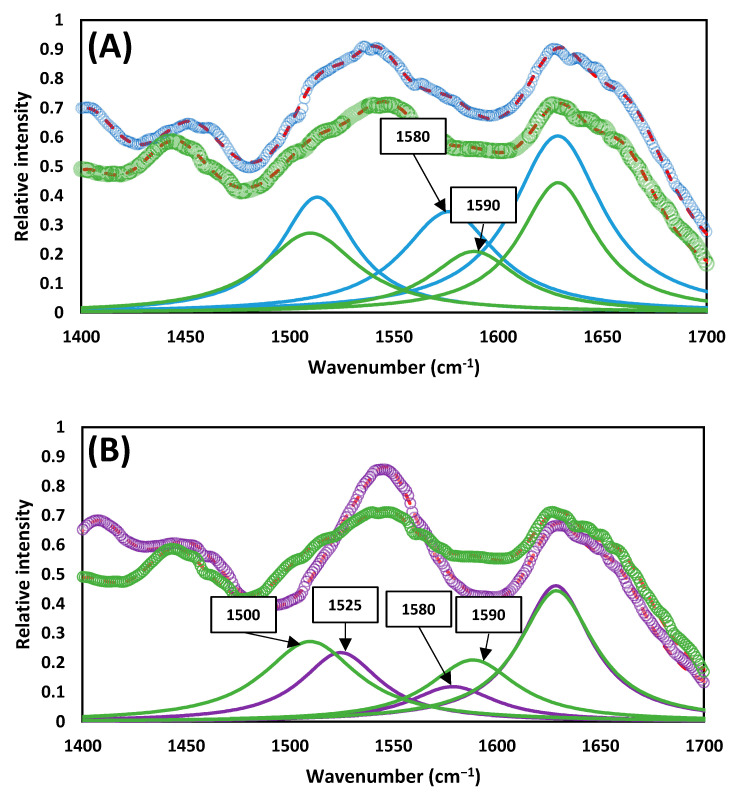
Comparison of the deconvoluted spectra with the fitted component bands at the range 1700 cm^−1^ and 1400 cm^−1^ of (**A**) the digested sludge after the TH-AD process (green) and the thermally pre-treated thickened activated sludge prior to its digestion in the TH-AD digester (blue) and (**B**) the digested sludge after the CAD process (purple) and the digested sludge after the TH-AD process (green).

After digestion, the intensity of the peaks at 1630 cm^−1^, 1580 cm^−1^, and 1510 cm^−1^ decreased slightly (see Figure 5A). A slight shift in position also occurred from 1580 cm^−1^ to 1590 cm^−1^. When comparing these peaks for the sludge digested by CAD and TH-AD processes (see Figure 5B), it can be clearly observed that there is a difference not only in peak position, but also in peak intensity, on the range between 1590 cm^−1^ and 1510 cm^−1^. This contrasts with the peak at 1630 cm^−1^ that did not suffer major changes.

## 4. Discussion

### 4.1. Performance of the Operation of TH-AD and CAD Digesters

The performance results of the CAD and TH-AD digesters are consistent with the ones reported by Valo et al. (2004) [39], Oosterhuis et al. (2014) [40], and Bougrier et al. (2006) [41], who used non-thickened, waste-activated sludge as a substrate. The increase in methane production and COD degradation in the TH-AD digester is attributed to the COD solubilization, as a result of the thermal pre-treatment stage (17.1% in this study). TAN yield differences could be attributed to a higher degradation of proteins after the TH-AD process [6,9]. A higher coloration on the liquid fraction of the digested sludge was also observed by Bougrier et al. (2007) [18], when pre-treating at 190 °C, suggesting the formation of Maillard reaction compounds.

As already commented, BMP assays were made to establish how much biodegradable organic matter remained in the digestates after the the CAD and TH-AD processes. The assays revealed that, since similar biodegradation and kinetics were obtained, increasing the HRT of the digesters will most probably not benefit one process over the other.

### 4.2. Macromolecular and Structural Study of the Sludges Produced by TH-AD and CAD Processes

CAD process effectively degraded proteins and carbohydrates. The observed increase in humic-like compounds could be explained by the humification process that occurs naturally during sludge storage and treatment, as a result of microbial activity [42]. This is supported by the increase in the peaks observed in the IR spectra of the digested sludge (between 3000 cm^−1^ and 3600 cm^−1^) in comparison with the untreated one, since one of the main characteristics of natural humic substances is the high presence of hydroxyl groups [43,44,45] in comparison to N related groups [46]. This is also consistent with Li et al. (2017) [47], who also observed broader peaks around the same area in fulvic and humic acids extracted from digested sludge. Liu et al. (2019) [48] also identified increased oxygen-containing functional groups after the digestion of waste-activated sludge. The same was observed by El Fels et al. (2014) [49] in the co-composting of sewage sludge and palm tree waste.

On the other hand, when thermal pre-treatment was applied, three superimposed phenomena occurred: the solubilization of macromolecules, the chemical transformation via Maillard and caramelization reactions, and the dilution of the sludge due to steam addition. The increase in the solubility of proteins and carbohydrates is a well-known fact [4,6,9]. However, solubilization alone cannot explain the 38% increase in CODs showed in Table 1.

Maillard and caramelization reactions are likely to occur due to the harsh conditions of the pre-treatment, leading to the formation of melanoidins and caramelans [9]. As seen in Figure 2C,D, humic-like compounds, formed due to the harsh conditions of the pre-treatment, increased considerably in both the soluble and particulate phases. This can be correlated with the increase in peaks at 1630 cm^−1^, associated with the early stage [30,31], and 1580 cm^−1^ and 1510 cm^−1^, associated with the intermediate and final stages of Maillard reactions [30,32,33,34,35,36,37,38]. The appearance of these peaks was described Mendez et al. (2014) [50] during the pre-treatment of microalgae; by Rodríguez-Abalde et al. (2013) [51] when applying thermal pre-treatment to slaughterhouse waste; by Yang et al. (2019) [14] during the preparation of artificial humic acid, and in our previous research dealing with the thermal pre-treatment of a synthetic substrate [16].

Further analysis of the IR spectra revealed that no prominent hydroxyl group was present, in comparison with humic-like compounds at the end of the CAD process. This was attributed to the occurrence of dehydration and condensation reactions as a part of the intermediate and final stages of Maillard reactions, suggesting an increased aromaticity. This reduction in hydroxyl groups was also observed by Ribeiro et al. (2001) [44] when drying humic substances for 1 h at 120 °C, by Yang et al. (2019) [14] in its preparation of artificial humic substances, by Vergnoux et al. (2011) [46] in the study of humic substances of burned soils, and by us in the thermally treated mixture of glucose and glycine [16]. This suggests that natural humic substances and artificial humic substances produced via thermal treatments may be differentiated by its hydration degree.

An often-overlooked phenomenon is the dilution of sludge in thermal pre-treatment. The CAMBI process injects steam directly into the hydrolysis reactor to increase its temperature. However, it also dilutes the sludge [5]. In this study, the sludge used was diluted around 46% (i.e., the total volume increased 46%), which must be considered when evaluating performances. This dilution explains the differences observed in total COD and volatile solids in Table 1 and the decrease in total proteins and carbohydrates observed in Figure 2C between the pre-treated and untreated sludge.

After digestion of the pre-treated sludge, carbohydrates and proteins were degraded. However, only a small amount of the total humic-like compounds formed at the pre-treatment step were degraded. Moreover, soluble humic-like compounds increased considerably. However, due to the complexity of the sludge, the quantity of the humic-like compounds that correspond to those formed during the pre-treatment step and how many were formed due to microbial activity cannot be identified. Lu et al. (2018) [20] reported a production of 0.258 g of humic acid g VS^−1^ at the thermal pre-treatment stage, which is very close to the amount obtained in this study. Moreover, the results obtained are in line with our previous research with a mixture of glucose/glycine pre-treated at the same conditions of time and temperature, where around 30% of COD of the original mixture was transformed into recalcitrant compounds that were not degraded in the anaerobic digestion stage [16].

Similarly, the peaks related with Maillard reaction products decreased only slightly after digestion (see Figure 4C,D). Moreover, the peak at 1580 cm^−1^ slightly changed its position to 1590 cm^−1^ (see Figure 5A). In summary, the peaks centered at 1630 cm^−1^, 1580 cm^−1^, and 1510 cm^−1^ are strongly related to the dissolved organic nitrogen, and therefore can be linked to the apparition of humic-like compounds after the pre-treatment stage.

Considering these results, it may be possible to infer the presence of recalcitrant compounds at the effluent of the wastewater treatment plant, since the liquid fraction of the digestate is often recirculated at the beginning of the treatment installation; moreover, melanoidins, which are recognized as part of the recalcitrant dissolved organic nitrogen [11], are poorly degraded in a typical activated sludge process [52,53,54] and in other nitrogen removal processes [54,55], being only effectively removed by an expensive and advanced oxidation process [54,56] or by the activation of persulfates [57]. Moreover, melanoidins would also be found at the place of sludge disposal; since half of these compounds are in suspension, the proportion that could increase depending on the type of polyelectrolytes used at the dewatering step, and their effect in sludge application, is still a matter of debate.

When comparing the spectrum of the sludge digested by the CAD process and the one digested by the TH-AD process (see Figure 5B), it can be clearly seen that there is a difference not only in peak position, but also in peak intensity, on the range between 1590 cm^−1^ and 1510 cm^−1^ associated with the intermediate and final stages of the Maillard reactions. This is in contrast with the peak at 1630 cm^−1^, which is associated with the early stages of Maillard reactions that did not suffer major changes. Early stages of Maillard reactions produce simpler and more biodegradable compounds in comparison to final stages. This would indicate that the peaks at 1590 cm^−1^ and 1510 cm^−1^ are the most important in relation to the recalcitrant compounds formed during the thermal pre-treatment step.

These results illustrate how and where the recalcitrant compounds are formed in a thermal pre-treatment process. Moreover, the presented results provide an insight on how the FTIR technique can help to identify these compounds and a possible way to differentiate them from natural humic substances—knowledge that may be very useful for future research in the field.

## 5. Conclusions

Thermal pre-treatment improves anaerobic digestion performance. However, it has the downside of producing compounds recalcitrant to anaerobic digestion. These compounds are an important part of the dissolved organic nitrogen affecting the downstream process because of their lower biodegradability. During this research, fresh and new information about the structure of such compounds was provided by following specific peaks in the IR spectra of sludge through each stage of the TH-AD process. The increase in two peaks located at 1590 cm^−1^ and 1510 cm^−1^ were strongly correlated with these recalcitrant compounds. These peaks are associated with a characteristic amide II bond that is a fundamental part of their structure and may explain its contribution to the dissolved organic nitrogen. A major difference from natural humic compounds was also observed in the lack of a prominent hydroxyl group. Recalcitrant compound formation is a serious drawback of the thermal hydrolysis pre-treatment, therefore it is imperative to advance in their understanding, monitoring, and treatment and/or possible usage.

## Figures and Tables

**Table 1:** Characterization of the substrates used with its respective standard deviation.

	Soluble COD (g L^−1^)	Total COD (g L^−1^)	Volatile Solids (g L^−1^)
Thermal pre-treated thickened activated sludge	54 ± 2	139 ± 12	84.6 ± 3.2
Thickened activated sludge	39 ± 3	221 ± 34	116 ± 18

**Table 2:** Results of the operations of CAD and TH-AD and its respective standard deviation.

	CAD	TH-AD
pH	8.4 ± 0.2	8.3 ± 0.4
VS degradation (%)	46.9 ± 3.3	49.5 ± 4.1
COD degradation (%)	41.8 ± 7.3	58.7 ± 5.4
CODs degradation (%)	23.8 ± 2.8	68.7 ± 3.5
Methane volumetric productivity (mL L digester^−1^ d^−1^)	230.3 ± 53.5	766.5 ± 88.2
Biogas composition (% methane)	62.7 ± 2.2	68.8 ± 2.1
Specific methane yield (mL CH_4_ L digester^−1^ g VSadded^−1^)	76.8 ± 17.8	225.4 ± 25.9
Color (Pt-Co units)	3250 ± 150	24800 ± 210
Total ammoniacal nitrogen in the digestate (TAN) (mg TAN L^−1^)	7440 ± 232	5280 ± 189
TAN yield (mg TAN g VS of thickened activated sludge ^−1^)	38.7	43.0
Volatile fatty acids/Alkalinity ratio (mg CH3COOH mg CaCO3^−1^)	0.19 ± 0.02	0.08 ± 0.01

## Data Availability

All data generated or analyzed during this study are included in this published article.

## Supplementary Materials

The following supporting information can be downloaded at: https://www.mdpi.com/article/10.3390/ijerph20010558/s1, Supplementary data 1: Figures; Figure S1. Biodegradability of the digestates of CAD and TH-AD digesters. Error bars indicate standard deviation between replicas; Figure S2. (a) FTIR of the untreated, undigested sludge, i.e., CAD digester feed (green lines); and effluent of CAD digester, i.e., anaerobically digested sludge in CAD digester (blue lines). The deconvoluted spectrum with the fitted component band is also shown for (b) the digested sludge and (c) the undigested sludge; Figure S3. (a) FTIR of the sludge before (green lines) and after (blue lines) thermal treatment, during TH-AD process, between the range of 4000 cm^−1^ and 2000 cm^−1^. The deconvoluted spectrum with the fitted component band is also shown for (b) the pre-treated sludge and (c) the untreated sludge and Table S1: Summary of the parameters of operation for CAD and TH-AD digesters.
