# Supplementary material for: Formation of Recalcitrant Compounds during Anaerobic Digestion of Thermally Pre-Treated Sludge: A Critical Macromolecular and Structural Study
Source: Int J Environ Res Public Health. 2022 Dec 29;20(1):558. doi: 10.3390/ijerph20010558 (PMC9819852; doi:10.3390/ijerph20010558)
Supplement: Supplementary file 1 [file ijerph-20-00558-s001.zip › ijerph-2059461-supplementary.pdf]

Supporting Information

Formation of recalcitrant compounds during anaerobic digestion of thermally pre-treated sludge: A macromolecular and a structural study

E. Ortega-Martínez[a], R. Chamy [a, b], D. Jeison [a]*

[a] Escuela de Ingeniería Bioquímica, Pontificia Universidad Católica de Valparaíso, Avenida Brasil 2085, Valparaíso, Chile.

[b] Núcleo Biotecnología Curauma, Pontificia Universidad Católica de Valparaíso, Avenida Universidad 330, Valparaíso, Chile.

*Corresponding author (e-mail address: david.jeison@pucv.cl).

Table S1. Summary of the parameters of operation for CAD and TH-AD digesters.

	CAD	TH-AD
Working Volume (L)	1	1
HRT (days)	33	25
Organic loading rate (g VS L^{-1} $days^{-1}$)	3.0	3.4
Stirring speed (RPM)	150	150
Stirring device	Orbital Agitator	Orbital Agitator
Operation time (days)	260	260

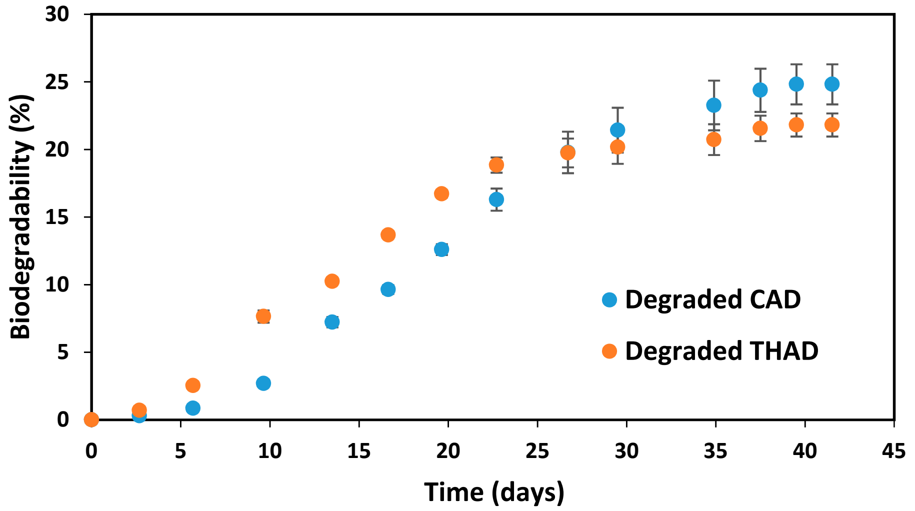

Figure S1. Biodegradability of the digestates of CAD and TH-AD digesters. Error bars indicate standard deviation between replicas.

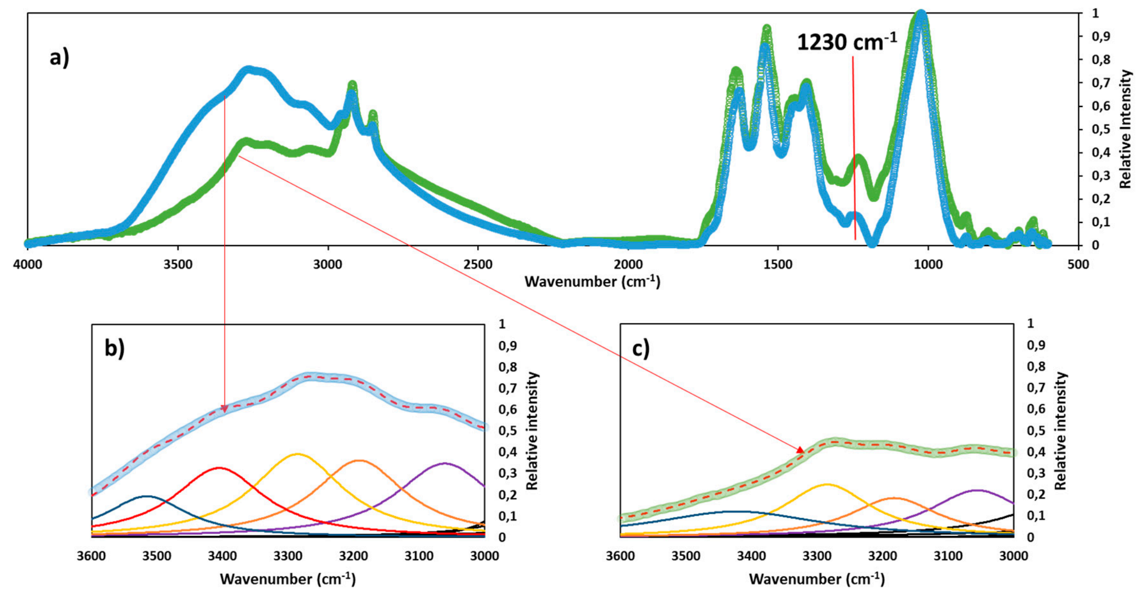

Figure S2. a) FTIR of the untreated, undigested sludge, i.e., CAD digester feed (green lines); and effluent of CAD digester, i.e., anaerobically digested sludge in CAD digester (blue lines). The deconvoluted spectrum with the fitted component band is also shown, for b) the digested sludge and c) the undigested sludge.

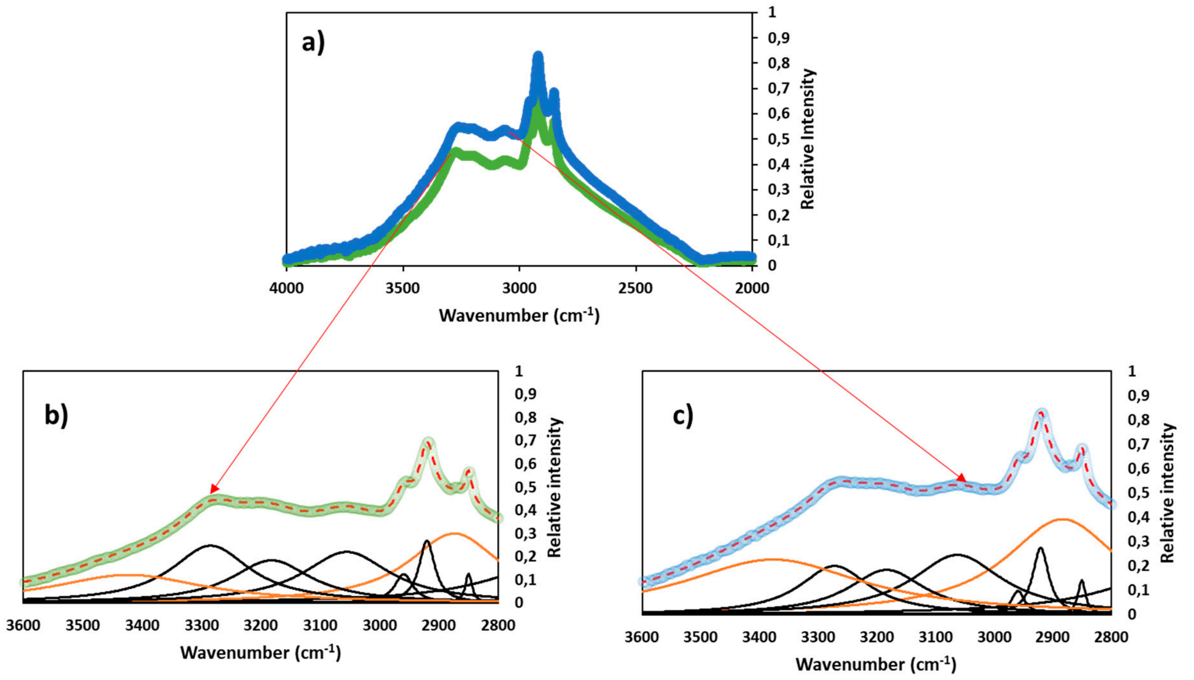

Figure S3. a) FTIR of the sludge before (green lines) and after (blue lines) thermal treatment, during TH-AD process, between the range of 4000 cm^{-1}. to 2000 cm^{-1}. The deconvoluted spectrum with the fitted component band is also shown, for b) the pre-treated sludge and c) the untreated sludge.
